# Novel, pathogenic insertion variant of GSDME associates with autosomal dominant hearing loss in a large Chinese pedigree

**DOI:** 10.1111/jcmm.18004

**Published:** 2023-10-20

**Authors:** Jingliang Cheng, Ting Li, Qi Tan, Jiewen Fu, Lianmei Zhang, Luquan Yang, Baixu Zhou, Lisha Yang, Shangyi Fu, Alora Grace Linehan, Junjiang Fu

**Affiliations:** ^1^ Key Laboratory of Epigenetics and Oncology, The Research Center for Preclinical Medicine Southwest Medical University Luzhou China; ^2^ Department of Pathology The Affiliated Huai'an No. 1 People's Hospital of Nanjing Medical University Huai'an China; ^3^ Department of Gynecology and Obstetrics Guangdong Women and Children Hospital Guangzhou China; ^4^ Department of Obstetrics The Affiliated Hospital of Southwest Medical University Luzhou China; ^5^ Department of Molecular and Human Genetics Human Genome Sequencing Center, Baylor College of Medicine Houston Texas USA; ^6^ School of Arts and Sciences New York University Shanghai Shanghai China

**Keywords:** genetics, GSDME, nonsyndromic hearing loss (NSHL), variant, whole‐exome sequencing (WES)

## Abstract

Nonsyndromic hearing loss (NSHL) is a genetically diverse, highly heterogeneous condition characterised by deafness, and Gasdermin E (*GSDME*) variants have been identified as directly inducing autosomal dominant NSHL. While many NSHL cases associated with GSDME involve the skipping of exon 8, there is another, less understood pathogenic insertion variant specifically found in Chinese pedigrees that causes deafness, known as autosomal dominant 5 (DFNA5) hearing loss. In this study, we recruited a large Chinese pedigree, conducted whole‐exome and Sanger sequencing to serve as a comprehensive clinical examination, and extracted genomic DNA samples for co‐segregation analysis of the members. Conservation and expression analyses for GSDME were also conducted. Our clinical examinations revealed an autosomal dominant phenotype of hearing loss in the family. Genetic analysis identified a novel insertion variant in *GSDME* exon 8 (GSDME: NM_004403.3: c.1113_1114insGGGGTGCAGCTTACAGGGTGGGTGT: p. P372fs*36). This variant is segregated with the deafness phenotype of this pedigree. The *GSDME* gene was highly conserved in the different species we analysed, and its mRNA expression was ubiquitously low in different human tissues. In conclusion, we have successfully identified a novel pathogenic insertion variant of *GSDME* in a Chinese pedigree that causes deafness, shedding light on the genetic basis of hearing loss within this specific family. Our findings expand the spectrum of known variants associated with GSDME‐related deafness and may further support both the underlying gain‐of‐function mechanism and functional associations of GSDME hearing loss variants.

## 1 INTRODUCTION

The Gasdermin E (*GSDME*) gene (OMIM: 608798), otherwise known to inversely correlate with the oestrogen receptor expression 1 (*ICERE‐1*) gene,^1^, ^2^ is located on chromosome 7p15.3. This *GSDME* gene includes 10 exons and encodes the 496 amino acids of the GSDME protein.^3^

Hearing loss is one of the most significant hereditary heterogeneous diseases^4^, ^5^ present in at least 1 out of every 500 newborns worldwide^6^; up to 60% of these cases have a genetic aetiology.^7^, ^8^ During the past two decades, tremendous progress has been made in the identification of causative genes in hereditary nonsyndromic hearing loss, or NSHL.^9^, ^10^ To date, a total of 124 NSHL genes have been identified, estimating 51 autosomal dominant (deafness autosomal dominant; DFNA) genes; 77 autosomal recessives (deafness autosomal recessive; DFNB) genes and 5 X‐linked (deafness X‐linked; DFNX; http://hereditaryhearingloss.org/) genes.

These play crucial roles in various functions of the ear that are essential for normal hearing. Such operations include cochlear fluid homeostasis, ion channel function, stereocilia morphology and function, synaptic transmission, and gene regulation. However, the precise function of some of these genes remains unclear. Among the autosomal dominant deafness genes, *GSDME* was extensively studied in 1998 in a Dutch family with NSHL, known as deafness autosomal dominant 5 (DFNA5, OMIM: 600994).^2^, ^3^ Deleting the 5 G‐triplets at the 3′‐end of the intron led to the skipping of exon 8. Consequently, a frameshift mutation occurred at amino acid 330, resulting in an aberrant stretch of 41 amino acids followed by a stop codon.^3^ This truncation of the protein leads to its premature termination and imparts cytotoxic activity.^11^

Since the Dutch study, only a limited number of families with *GSDME* variants associated with hereditary NSHL have been documented in current scientific literature.^12^, ^13^, ^14^, ^15^, ^16^, ^17^, ^18^ Although the age of initial onset varies, ranging from 11 to 50 years, the hearing loss function in these families exhibits similar characteristics. These presentations include progressive hearing loss, with higher frequencies being affected first and the remaining frequencies later; nonsyndromic and sensorineural deafness. Sensorineural deafness, a neurologically related hearing loss that is the likely result of environmental and genetic interaction, involves damage to the cochlear receptors of the inner ear, the auditory nerve fibres that connect the inner ear to the brain, or specific brain regions responsible for processing sound information. Prolonged listening to noise is one example of a potential contributing factor. Moreover, many types of sensorineural hearing loss are exclusively linked to highly penetrant genetic causes where the environment is not contributive. This disease is attributed to variants that affect the gene represented in this study. The majority of reported variants in the *GSDME* gene result in the skipping of exon/intron 8 at the messenger (mRNA) level, resulting in a frameshift and premature truncation of the GSDME protein.^19^ In our study, we identified a novel variant of *GSDME* in a large Chinese family with nonsyndromic sensorineural hearing impairment. To the best of our knowledge, this is the first report describing a *GSDME* variant with a significant nucleotide insertion.

## 2 METHODS

### 2.1 Pedigree and sample collections

**FIGURE 1:**
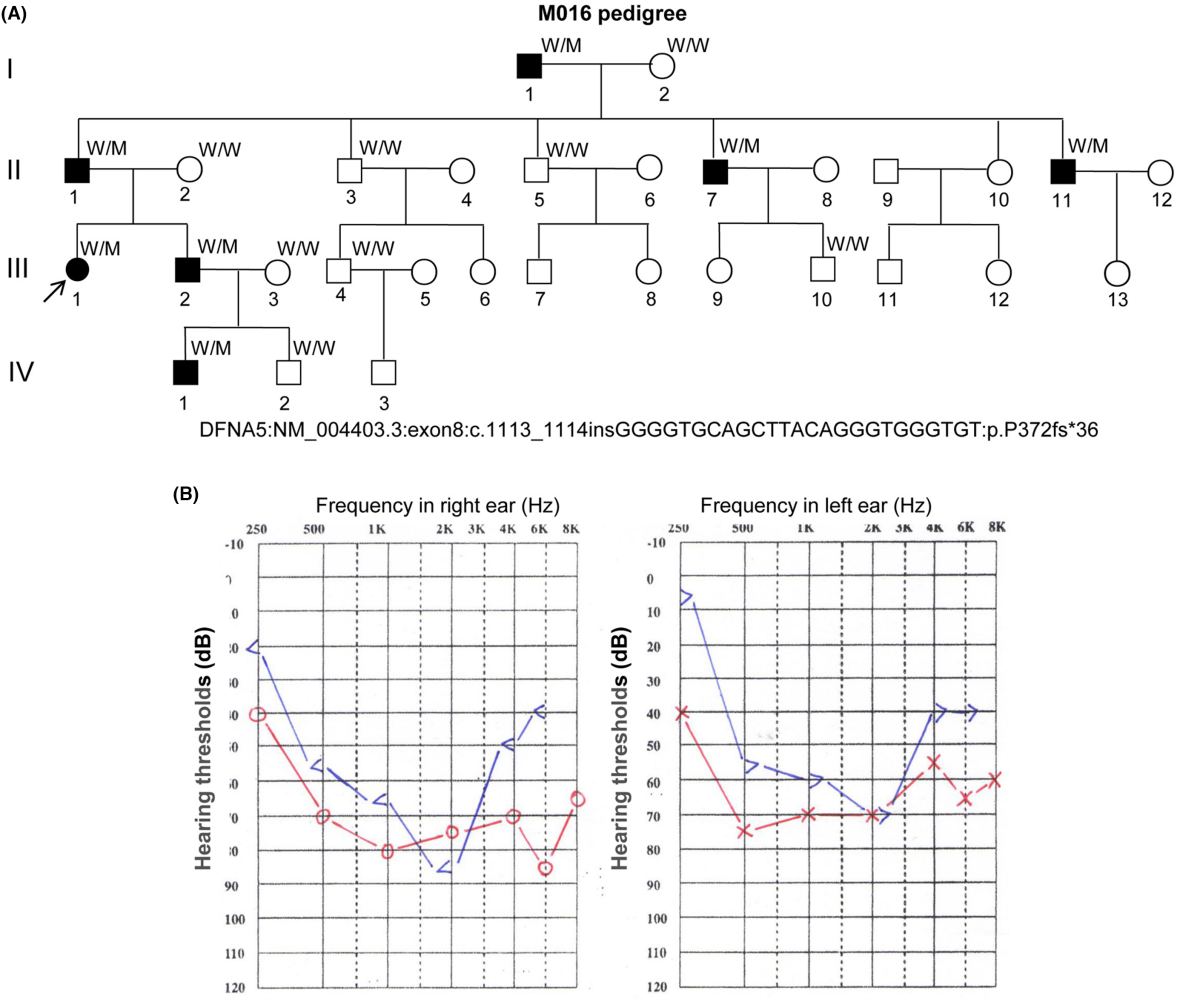
The M016 pedigree presents autosomal dominant nonsyndromic sensorineural deafness. (A) A large pedigree with autosomal dominant nonsyndromic sensorineural deafness. ↗, Proband; M, Mutation allele; W, Wild‐type allele. Information for *GSDME* variant: NM_004403.3:exon8:c.1113_1114insGGGGTGCAGCTTACAGGGTGGGTGT:p.P372fs*36. (B) Pure‐tone audiograms of the proband (III:1). The audiograms present bilateral severe hearing loss across all frequencies at the age of 11 years. Red and blue lines indicate air and bone conduction, respectively. In air conduction, the right and left ears are indicated by “o” and “x”; in bone conduction, the right and left ears are indicated by “<” and “>”. “dB”, decibel.

In our study, we enrolled a four‐generation Chinese family consisting of 30 members, including a proband and six additional patients with diagnosed hearing loss (Figure 1A, pedigree III: 1, arrow, molecular no. M016). The use of a questionnaire allowed us to identify a dominant inheritance pattern; the pedigree's proband was clinically examined according to the criteria recommended by NSHL.^7^ Audiograms in the proband and family members were constructed using pure‐tone audiometry with different frequencies of 0.25, 0.5, 1, 2, 3, 4, 6 and 8 kHz. The pure‐tone threshold audiometry included both air conduction audiometry and bone conduction audiometry. During conduction audiometry testing, the initial frequency used started at 0.25 kHz. After the patient heard the sound, the sound intensity was lowered step‐wise at intervals of 5 hearing thresholds of decibels (dB) until the patient could no longer identify the sound. Subsequently, another test was performed, where audiometry was repeated until the exact hearing threshold was determined. The sound intensity increased step‐wise (an increase of 5 dB every time), and the hearing threshold at other frequencies was tested similarly. It is important to note that intermittent tones must also be used to avoid auditory fatigue. The bone conduction test followed a similar process to the air conduction audiometry.

For this study, we collected peripheral blood from patients and their family members. Genomic DNA (gDNA) was extracted from leukocytes using protease K and phenol/chloroform.^20^, ^21^ The research protocol was approved by the Ethical Committees of Southwest Medical University.

### 2.2 Whole‐exome sequencing (WES) and variation analysis

The proband (molecular no. M016 [III:1]) and other three patients (molecular Nos. M017 [II:1], M020 [I:1], and M285 [II:11]) were screened by WES using an Illumina HiSeq X Ten Next Generation Sequencing platform (NGS) (Illumina). The gene captured up to 200 × depth and had a coverage of over 90%. For the identification of variants, the sequencing data was analysed; the original data was FASTQ formatted, which was aligned to hg19 using a Burrows–Wheeler Alignment (BWA) aligner (BWA version 0.5.9). Following recalibration and local realignment, the refined sequencing underwent variant calling using the Atlas2 toolkit to filter out common polymorphisms (an allele frequency of >0.5%). Variant annotation and pathogenicity prediction were applied for novel variants. Then, the loci were screened based on clinical symptoms and genetic patterns.

### 2.3 Polymerase chain reaction (PCR) and Sanger sequencing analysis

For variant validation, we designed *GSDME* primers, namely GSDME‐L: 5′ CTTGGTCTCCAGCTGTGTCA 3′ and GSDME‐R: 5′ GAGTAGGGAGGCTTGTGCTG 3′, based on *GSDME* variant in exon8, NM_004403.3. The templates for gDNA from all indicated individuals were amplified by PCR using a traditional amplification machine (Life Technologies) and subjected to Sanger sequencing analysis using an ABI‐3500DX sequencer.^22^ The amplified PCR product contained 459 bp and primer GSDME‐L was used for Sanger sequencing.

### 2.4 Conservation and expression analysis for GSDME

Conservation analysis for GSDME was conducted at the National Center for Biotechnology Information (NCBI) for different species (https://www.ncbi.nlm.nih.gov/Structure/cdd/wrpsb.cgi?INPUT_TYPE=live&SEQUENCE=NP_004394.1).^23^, ^24^ Expression analysis of *GSDME* mRNA was conducted for different normal tissues in humans on the Human Protein Atlas (HPA) project (https://www.proteinatlas.org/ENSG00000105928‐GSDME/tissue).^25^, ^26^

## 3 RESULTS

### 3.1 Pedigree recruitment and clinical findings

The M016 pedigree (Figure 1A) represents 30 members spanning 4 generations and was recruited when the proband was 26 years old. According to the proband, she experienced hearing loss at the age of 7 and began requiring a hearing aid to assist with sound perception. Over time, her hearing ability gradually deteriorated. According to the result of pure‐tone audiometry, the proband's hearing impairment declined with age. The hearing loss of higher frequencies was the most severe, as displayed by a negative sloping audiometric configuration (Figure 1B). The hearing loss disorder was determined to be caused by a genomic abnormality that affects the auditory sensory system or neural components of the auditory pathway, resulting in hearing dysfunction. However, it is not accompanied by other systemic abnormalities. Results from our other clinical examinations, such as middle ear examination, mastoid examination and vestibular function tests, showed no other inner ear‐related abnormalities; with the exception of deafness, the patient was phenotypically normal. None of the patients claimed vestibular symptoms, and the proband's father and brother also display the same clinical symptoms. Upon tracing the pedigree tree, it was observed that the proband's nephew had natural hearing at birth but developed deafness at the age of 8. Hearing loss symptoms were first reported at ages 7–30, manifesting themselves as progressive and nonsyndromic. In all of the patients in this family, the hearing loss worsened with age, yet none of the individuals are currently completely deaf. Thus, in this family, the inheritance is diagnosed as NHSL autosomal dominant.

### 3.2 Identification of the novel GSDME variant

**FIGURE 2:**
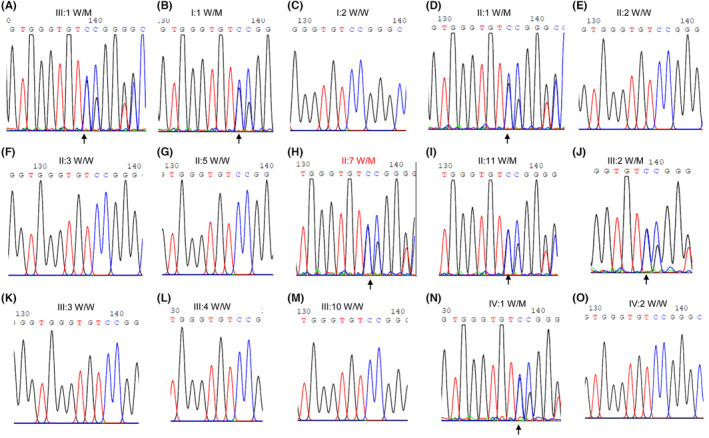
The *GSDME* sequence chromatogram of the pedigree members. (A) The *GSDME* variant of the proband is verified by Sanger sequencing. (B–O) The different family members are indicated by Sanger sequencing.

Fifteen blood samples were taken for DNA extraction from the patients, who were all alive when the study was conducted. WES was conducted for the proband (Figure 1A,III:1), proband's father (Figure 1A,II:1), grandfather (Figure 1A,I:1), and uncle (Figure 1A,II:11). High‐quality NGS data were obtained. The results revealed that all of the affected individuals with hearing loss presented a 25‐bp heterozygous insertion (GGGGTGCAGCTTACAGGGTGGGTGT) in exon 8 of the *GSDME* gene (NM_004403.3: c.1113_1114). Sanger sequencing verified the heterozygous variant for III:1, I:1, II:1 and II:11 (Figure 2A,B,D,I). The proband's brother (Figure 1A,III:2, Figure 2J) demonstrated the same *GSDME* variant, whereas his wife presented the wild‐type of *GSDME* (Figure 1A,III:3, Figure 2K). However, their son exhibited the GSDME variant and later developed hearing loss, which worsened with age (Figure 1A,IV:1, Figure 2N).

This variation was absent in the 100 ethnically normal controls or the 1000 Human Genome Project, Human Gene Mutation Database (HGMD), ExAC and gnomAD, Chinese Millionome Database and ClinVar databases; thus, we concluded that this was a novel variant. This variant was submitted to the ClinVar database with the access number SUB13488920. The confirmation of co‐segregation in this pedigree further indicates the possible role of this novel *GSDME* variant in the pathogenesis of hearing loss.

### 3.3 Variant c.1113_1114insGGGGTGCAGCTTACAGGGTGGGTGT: p.P372fs*36 for *GSDME* is pathogenic

**FIGURE 3:**
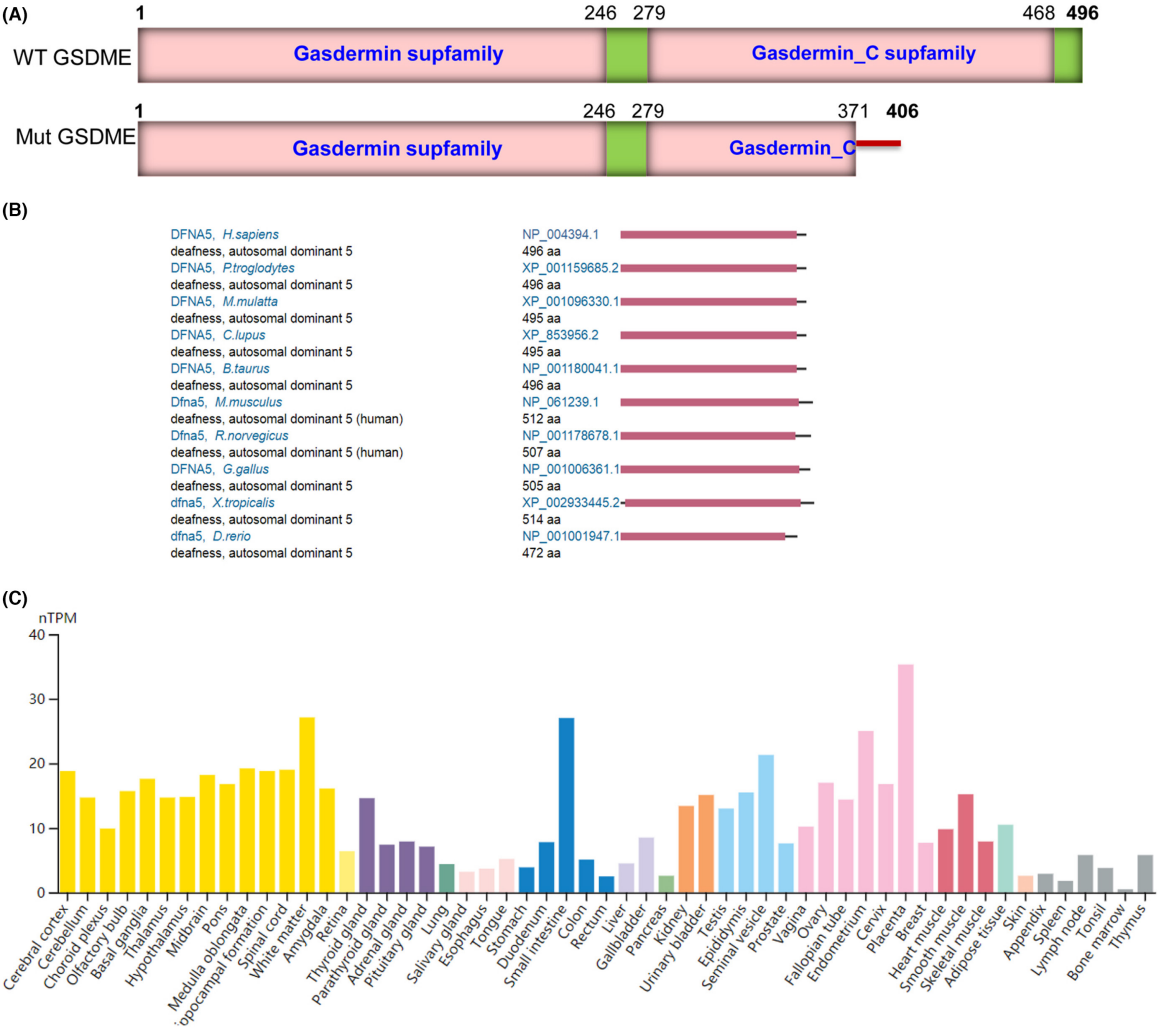
The structure of both wild‐type and variant forms; conservation and expression of *GSDME*. (A) Schematic representation of both wild‐type and variant forms of *GSDME*. (B) The conservation of *GSDME*. (C) The mRNA expression of *GSDME*.

A 25‐nucleotide insertion (GGGGTGCAGCTTACAGGGTGGGTGT) occurred at the nucleotide position 1113 (NM_004403.3), thereby resulting in a mutation from the “T” position to GGG (G) at amino acid position 372. The insertion by CCG (P) was followed by an additional 34 amino acids and a stop codon (*, TAG). More specifically, the insertion resulted in the creation of a novel stop codon after the synthesis of 35 additional amino acids (Figure 3A). The variant truncated 125 amino acids at the C‐terminal of the GSDME protein, calculating one‐fourth of the full‐length protein. Based on the evidence of PVS1 criteria, which suggests the variant is predicted to induce a loss of function (LOF) through a known disease mechanism, the variant c.1113_1114insGGGGTGCAGCTTACAGGGTGGGTGT:p.P372fs*36 is hypothesised to be pathogenic. This assessment follows the guidelines set forth by the American College of Medical Genetics and Genomics (ACMG) and the Association for Molecular Pathology Guidelines (AMP).^27^, ^28^

### 3.4 Co‐segregation analysis

Co‐segregation analysis indicated that, including patient IV:1, all patients had the same heterozygous variant (Figure 2), whereas other members with normal hearing had the wild‐type allele of the *GSDME* gene. The family member IV:1 is 12 years old, and the last clinical visit indicated normal hearing at 6 years old. Subsequently, the parents identified this abnormality when the boy was 8 years old and verified it using pure‐tone audiometry (data not shown), demonstrating either Sanger sequencing is correct or that co‐segregation is consistent (Figure 2N,IV:1).

### 3.5 Conservation and expression analyses of GSDME

By conservation analysis, the *GSDME* gene was found to be highly conserved in chimpanzees, rhesus, monkeys, chickens, cows, dogs, mice, rats, zebrafish and frogs, with Gasdermin domains (Figure 3B).

The results of the HPA database, which analysed 55 tissue types, indicated that the expression of *GSDME* mRNA is ubiquitously low across various tissues. Among the samples analysed, the highest expression was observed in the sample placenta with 35.3 normalised transcripts per million (nTPM), followed by white matter with 27.1 nTPM, and the small intestine with 27 nTPM. Conversely, the lowest expression in bone marrow was 0.5 nTPM (Figure 3C). However, no cochlea data on *GSDME* mRNA expression is available. Previous studies have reported that the gene is highly expressed in the cochlea of mice; however, the function of this gene is still unclear.^29^ Nevertheless, based on conservation and expression analyses, GSDME was found to play a critical role in different tissues and species.

## 4 DISCUSSION

The GSDME protein is a member of the Gasdermin superfamily, characterised by a necrotic‐inducing N‐terminal domain. GSDME‐N (amino acids 1 ~ 270) is self‐inhibited by the C‐terminal domain of GSDME‐C (amino acids 271 ~ 496). When the linker of these two domains is cleaved by the killer cell granzyme B (GzmB) or the apoptotic protease caspase‐3,^30^, ^31^, ^32^ the released GSDME‐N fragment partakes in the cell death pathway by forming pores in the plasma and mitochondrion membranes. Thus, GSDME not only plays a role in nonsyndromic sensorineural hearing loss but also in tumour biology as a suppressor gene^1^, ^33^, ^34^, ^35^ by pyroptosis activation, enhancing antitumour immunity. An uncleavable or pore‐defective GSDME protein was not found to be tumour‐suppressive.^35^

Since the identification of the *GSDME* variant in 1998, which encompasses ten exons, several variants carrying gain‐of‐function pathogenicity have been associated with the skipping of exon 8 at the mRNA level, leading to a form of NSHL known as DFNA5 (OMIM #600994).^36^, ^37^ These variants were previously localised either on the flanks or the interior of exon 8. Mechanically, this out‐of‐frame skipping in exon 8 gives rise to a C‐terminal truncated protein with constitutively active necrosis. Not all *GSDME* variants cause hearing loss,^38^ but late‐onset progressive NSHL cannot be neglected.^39^

In our study, we successfully identified a novel insertion variant of *GSDME* in exon 8 (GSDME: NM_004403.3: c.1113_1114insGGGGTGCAGCTTACAGGGTGGGTGT: p.P372fs*36) that segregated with the occurrence of hearing loss of the affected family. For example, family member IV:1 first showed normal hearing, but the same NSHL phenotype was later found when the boy was 8 years old and began experiencing deafness. A wild‐type variant of *GSDME* was found in family member IV:2 at 17+ weeks of pregnancy through WES of amniotic fluid DNA in March 2019 (data not shown), verified by Sanger sequencing after birth (Figure 1A,IV:2 and Figure 2O), and presented normal hearing. This variant may not produce the potential aberrant splicing since there has been no new typical splicing structure (intron‐ag/TG…exon…AG/gt‐intron) at flanking sequences. To further support our prediction, we collected blood samples from patients and normal members in this pedigree to conduct reverse transcription (RT)‐PCR, but the amplification failed as the mRNA level of *GSDME* was very low (data not shown).

As of 4 February 2023, a total of 13 *GSDME* variants associated with DFNA5 have been reported in the HGMD: two missense/nonsense variants, seven splicing variants, two small deletions, one gross insertion/duplication (duplication of 99 bp) and one complex rearrangement. A majority of the reported variants cause the skipping of exon/intron 8 at the mRNA level, resulting in a frame shift for a prematurely truncated GSDME protein. To the best of our knowledge, the identified variant *GSDME*: NM_004403.3: exon 8: c.1113_1114insGGGGTGCAGCTTACAGGGTGGGTGT: p.P372fs*36 is the first reported insertion variant for *GSDME* that directly led to hearing loss, expanding the current variant spectrum. As a result of our findings, we submitted this variant to the ClinVar database with the accession number SUB13488920. Our results may further support the gain‐of‐function mechanism that associates the GSDME variants with hearing loss owing to the loss of the constitutively active C‐terminus.

## 5 CONCLUSION

In this study, we have successfully identified a novel insertion variant in exon 8 of the *GSDME gene* (GSDME: NM_004403.3: c.1113_1114insGGGGTGCAGCTTACAGGGTGGGTGT: p.P372fs*36) associated with hearing loss in a large Chinese pedigree family, thereby expanding the variant spectrum. Our findings may also support the gain‐of‐function mechanism that associated the *GSDME* variant with hearing loss (DFNA5). This study could have a potential value in China's current gene testing strategy, eventually benefiting genetic counselling and disease prevention.

## AUTHOR CONTRIBUTIONS

**Jingliang Cheng:** Funding acquisition (equal); investigation (equal); writing – original draft (equal). **Ting Li:** Investigation (equal). **Qi Tan:** Investigation (equal). **Jiewen Fu:** Investigation (equal); methodology (equal). **Lianmei Zhang:** Resources (equal). **Luquan Yang:** Resources (equal). **Baixu Zhou:** Investigation (equal). **Lisha Yang:** Investigation (equal). **Shangyi Fu:** Investigation (equal); writing – review and editing (equal). **Alora Grace Linehan:** Writing – review and editing (equal). **Junjiang Fu:** Conceptualization (equal); funding acquisition (equal); project administration (equal); supervision (equal); writing – original draft (equal); writing – review and editing (equal).

## FUNDING INFORMATION

This study was supported by the Joint Research Foundation of Luzhou City and Southwest Medical University (no. 2018LZXNYD‐YL01) and was partially funded by the National Natural Science Foundation of China (nos. 81672887, 31701087 and 30371493).

## CONFLICT OF INTEREST STATEMENT

The authors have no conflicts of interest to declare.

## ETHICS STATEMENT

The study was approved by the Ethical Committee of Southwest Medical University for human studies and written informed consent was obtained from participants. All written informed consents and procedures adhered to the tenets of the Declaration of Helsinki.

## PATIENT CONSENT

Obtained.

## Data Availability

The data that support the findings of this study are available from the corresponding authors upon reasonable request.
